# Azilsartan compared to ACE inhibitors in anti-hypertensive therapy: one-year outcomes of the observational EARLY registry

**DOI:** 10.1186/s12872-016-0222-6

**Published:** 2016-03-08

**Authors:** Anselm K. Gitt, Peter Bramlage, Sebastian A. Potthoff, Peter Baumgart, Felix Mahfoud, Hartmut Buhck, Martina Ehmen, Taoufik Ouarrak, Jochen Senges, Roland E. Schmieder, for the EARLY Registry Group

**Affiliations:** Institut für Herzinfarktforschung, Bremser Strasse 79, 67063 Ludwigshafen, Germany; Herzzentrum Ludwigshafen, Medizinische Klinik B, Ludwigshafen, Germany; Institut für Pharmakologie und präventive Medizin, Mahlow, Germany; Universitätsklinikum Düsseldorf, Klinik für Nephrologie, Düsseldorf, Germany; Clemens-Hospital Münster, Klinik für Innere Medizin I, Münster, Germany; Universitätsklinikum des Saarlandes, Klinik für Innere Medizin III, Homburg/Saar, Germany; MedCommTools, Medical-scientific consultancy, Hannover, Germany; Takeda Pharma Vertriebs GmbH, Berlin, Germany; Universitätsklinikum Erlangen, Medizinische Klinik 4, Schwerpunkt Nephrologie / Hypertensiologie, Erlangen, Germany

**Keywords:** Azilsartan medoxomil (AZL-M), Hypertension, Real world, Effectiveness, Safety

## Abstract

**Background:**

Azilsartan medoxomil (AZL-M), has been demonstrated to be more effective than the other sartans currently in use; however, there is insufficient information available comparing it with ACE-inhibitors. Therefore, we aimed to compare the efficacy, safety, and tolerability of AZL-M with that of ACE-inhibitors in a real life clinical setting.

**Methods:**

The EARLY registry is a prospective, observational, national, multicentre registry with a follow-up period of 12 months. There were two principal objectives: 1) documentation of the achievement of target BP values set according to recent national and international guidelines, and 2) description of the safety profile of AZL-M.

**Results:**

A total of 3 849 patients with essential arterial hypertension were recruited from primary care offices in Germany. Patients who initiated monotherapy at baseline comprising either AZL-M or an ACE-inhibitor were included at a ratio of seven to three. Results demonstrated that a blood pressure target of <140/90 mmHg was achieved by a significantly greater proportion of patients in the AZL-M group (61.1 %) compared with the ACE-inhibitor group (56.4 %; *p* < 0.05; OR, 1.21; 95 % CI, 1.03–1.42), with this finding maintained after adjusting for differences in baseline characteristics. AZL-M appeared to have an equivalent safety profile to the ACE-inhibitors, with a similar incidence of adverse events in the two patient groups (*p* = 0.73).

**Conclusions:**

These data add to the results of previous randomized controlled clinical trials suggesting that, compared with other agents that target the renin–angiotensin system, AZL-M provides statistically significant albeit small improvements in blood pressure control.

**Electronic supplementary material:**

The online version of this article (doi:10.1186/s12872-016-0222-6) contains supplementary material, which is available to authorized users.

## Background

Despite the availability of many safe and effective antihypertensive drugs, target blood pressure (BP) is only obtained in approximately 20 % of hypertensive patients in Germany [1]. It has been suggested that achieving a reduction in mean systolic BP (SBP) on the order of 2 mmHg can result in a 10 % reduction in the incidence of fatal stroke and a 7 % decrease in death due to ischaemic heart disease or other vascular causes [2]. This indicates the importance of developing more effective drugs for controlling hypertension.

Angiotensin converting enzyme (ACE) inhibitors are known to be effective in lowering BP, working by inhibiting the enzyme responsible for converting angiotensin I to angiotensin II in the renin–angiotensin system (RAS). However, they have also been associated with side effects such as a persistent cough and, less commonly, angioedema [3, 4]. Angiotensin receptor blockers (ARBs) are a more recently introduced class of antihypertensive drugs that also target the RAS, binding to the angiotensin receptor1 (AT1 receptor) for angiotensin II [5]. ARBs have demonstrated similar or improved efficacy for reducing BP but appear to be more tolerable, with fewer side effects such as coughing reported [5], although no significant differences in the occurrence of drug-related adverse events (AEs) have been noted [6–8]. There are a number of different ARBs used to treat hypertension, all working via the same mechanism but displaying different efficacies [9]. Azilsartan medoxomil (AZL-M) is the most recently approved ARB [10], and has demonstrated the capacity to reduce BP to a greater extent than olmesartan, which is considered to be the most potent of the sartans used clinically [11, 12].

A recent phase III trial compared the efficacy of AZL-M with the ACE-inhibitor Ramipril [13]. This comparison is particularly valuable as ramipril is considered to be a benchmark antihypertensive drug that has been shown to be highly effective in lowering BP [14, 15]. In a randomized clinical trial AZL-M was found to be more effective in lowering BP in comparison to ramipril; furthermore, patients in the ARB group experienced fewer AEs leading to discontinuation of treatment [13].

Based on these data, an improvement in BP control might be expected from using AZL-M in clinical practice. The EARLY “*Treatment with Azilsartan Compared to ACE*-*Inhibitors in Anti*-*Hypertensive Therapy*” registry was designed to document BP control and its impact on cardiovascular and renal events during a 12 month follow-up period in patients administered either AZL-M or an ACE-inhibitor at baseline [16].

## Methods

The EARLY registry is a prospective, observational, national, multicentre registry with a follow-up period of 12 months. Follow-up visits after 6 and 12 months were conducted by the sites. Details of the aims and design of the study protocol have been published previously [16, 17]. In short, patients with arterial hypertension having either no anti-hypertensive treatment prior to inclusion or a prior non-RAS based antihypertensive monotherapy and starting treatment on either AZL-M or any ACE inhibitor monotherapy in a ratio of 7 (AZL-M) to 3 (ACE-inhibitor) were documented. Treatment decisions were at the physician’s discretion, and groups were not randomized.

The protocol was approved by the independent international ethics committee in Freiburg, and the ethics committee of the State Medical Council of Rheinland-Pfalz, Germany. All patients enrolled in the registry provided written informed consent.

### Objectives

Establishment of the registry had two primary objectives: 1) documentation of the achievement of target BP values set according to recent national and international guidelines [18], and 2) description of the safety profile of AZL-M. Further secondary objectives are listed in the protocol published previously [16] and include the 1) absolute and relative BP reduction with antihypertensive treatment over the duration of one year 2) documentation of the adherence to guidelines for the diagnosis and treatment of hypertension in ambulatory care 3) persistence understood as the mean duration of monotherapy and/or AZL-M based combination therapy during follow-up 4) documentation of adverse events 5) prospective documentation of cardiovascular and renal events.

### Selection of sites and patients

The registry was established in primary care offices in Germany. Centres were selected from a database maintained at the Institut für Herzinfarktforschung, Ludwigshafen. The centres were chosen with the aim of obtaining data that is representative of current hypertension treatment in Germany.

Patients over 18 years of age with essential arterial hypertension were included on a consecutive basis [18], given that they had provided written informed consent and fulfilled the following two criteria: 1) they had either no antihypertensive treatment prior to inclusion or were on a non-RAS based antihypertensive monotherapy, and 2) monotherapy consisting of AZL-M or an ACE-inhibitor was initiated at baseline. Patients were excluded from participation if they 1) received antihypertensive drugs for an indication other than hypertension (e.g. beta blockers or diuretics for heart failure); 2) had a history of alcohol, drug abuse, or illegal drug addiction; 3) had a life expectancy of less than one year; 4) were pregnant or breast feeding; or 5) were participating in other trials or registries. Moreover, patients with contraindications as to the summary of product characteristics of any of the drugs being prescribed were excluded.

### Statistics

Reasons for choosing a 7 (AZL-M) to 3 (ACE-inhibitor) ratio and justification for the sample size have been published previously [16]. Continuous variables were analysed using descriptive statistics (absolute numbers, means plus standard deviations (SD), or medians with 25^th^ and 75^th^ percentiles) as appropriate. Categorical data were described by the number (n) and percentage (%) of subjects in each category. Comparisons between treatment groups were performed using Pearson’s chi-squared test for categorical variables, or the Mann–Whitney-Wilcoxon test for continuous measurements.

To assess differences in BP between groups that differed at baseline, two multivariate models were used (Table 2). Model 1 provided adjustments for SBP/diastolic BP (DBP) at baseline; while model 2 additionally took into account whether the hypertension was newly diagnosed or established, age, gender, and diabetes. *P*-values ≤ 0.05 were considered significant. All given *p*-values are the results of two-sided tests. Statistical analysis was performed using the SAS 9.3 software (SAS Institute, Inc., Cary, NC, USA).

## Results

### Patient characteristics at baseline

**Fig. 1:**
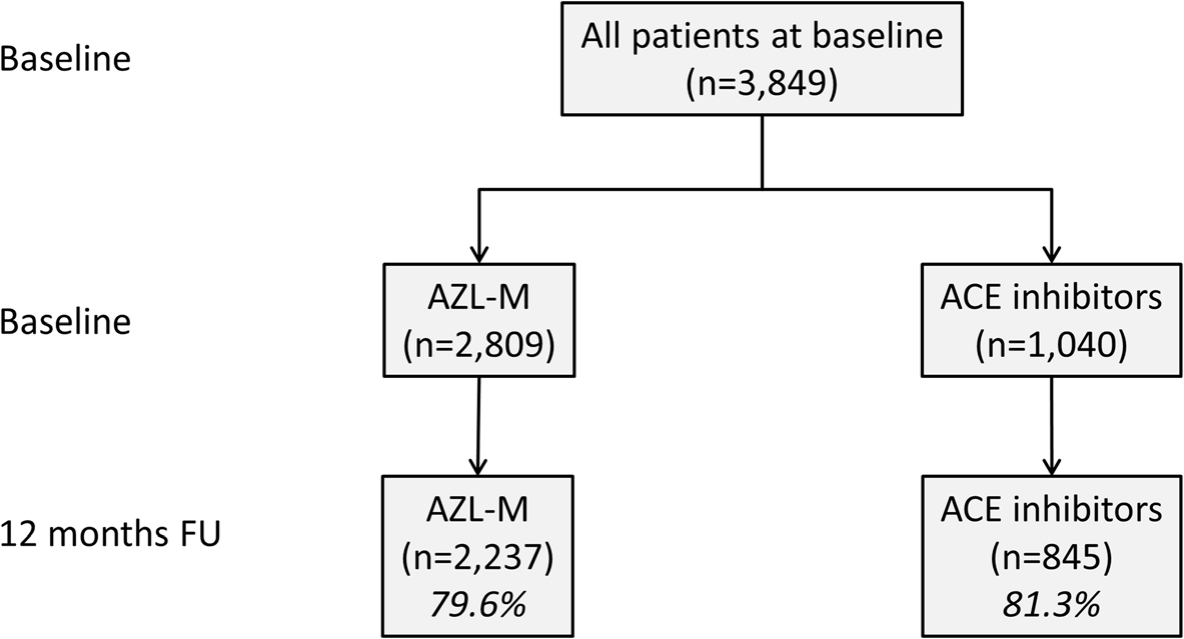
Patient flow chart

The EARLY registry enrolled a total of 3 849 patients (Fig. 1) in 509 sites. Of these, 2 809 (73.0 %) were treated with AZL-M (mean dose 41.4 ± 21.3 mg), with 1 040 patients (27.0 %) receiving an ACE-inhibitor (mean dose 7.6 ± 11.2 mg), mainly ramipril (889 patients, 85.5 %), reflecting the planned enrolment ratio of 7:3. Baseline characteristics of the patients are given in Table 1. The mean age of the overall population was 59.4 years with slightly older patients in the AZL-M than in the ACE-inhibitor group; there were also marginally more females (47.9 % vs. 43.8 %). Mean body weight did not vary significantly between treatment groups. The proportion of total patients with a new diagnosis was 36.9 %, with the remainder having established hypertension. There were fewer newly diagnosed patients in the AZL-M group (34.2 % vs. 43.9 %), and those with established hypertension had a longer mean time since diagnosis in comparison to the ACE-inhibitor group (67.2 ± 65.3 months vs. 57.7 ± 60.9 months; *p* < 0.001). Baseline office BP measurements revealed that only 6.1 % of all patients had SBP/DBP below 140/90 mmHg. The most frequent comorbidities of the patients are also given in Table 1, where it can be seen that diabetes and coronary artery disease (CAD) were the most prevalent. While the prevalence of CAD was slightly higher in the ACE-inhibitor group, those of the other comorbidities were similar.

**Table 1:** Baseline characteristics of patients with or without 12 months follow-up

	Total patients (*n* = 3849)	Without 12 month follow-up (*n* = 767)	With 12 month follow-up(*n* = 3 082)	*p*-value w vs. w/o follow-up	AZL-M (*n* = 2 809)	ACE-inhibitor (*n* = 1 040)	*p*-valueAZL-M vs. ACE-inhibitor
Age, years	59.4 ± 13,0	58.8 ± 13.4	59.6 ± 12.9	0.29	60.1 ± 12.6	57.7 ± 13.9	<0.0001
Female, %	46.8	41.5	48.1	<0.001	47.9	43.8	<0.05
Body weight, kg	83.3 ± 15.6	84.5 ± 15.8	83.0 ± 15.6	<0.05	83.4 ± 15.8	83.1 ± 15.2	0.94
Hypertension							
Newly diagnosed, %	36.9	34.7	37.4	0.16	34.2	43.9	<0.0001
Established, months	65.0 ± 64.4	65.3 ± 70.8	64.9 ± 62.7	0.49	67.2 ± 65.3	57.7 ± 60.9	<0.001
Office SBP, mmHg	159.3 ± 17.1	159.6 ± 17.9	159.3 ± 16.9	0.67	160.0 ± 17.4	157.6 ± 16.1	<0.0001
Office DBP, mmHg	93.5 ± 10.5	93.3 ± 10.8	93.5 ± 10.4	0.60	93.8 ± 10.6	92.7 ± 10.2	<0.01
B*P* < 140/90 mmHg, %	6.1	6.8	5.9	0.34	6.2	5.7	0.60
AZL-M treatment, %	73.0	74.6	72.6	0.27	100.0	0.0	
ACE-inhibitor treatment, %	27.0	25.4	27.4	0.27	0.0	100.0	
Comorbidity							
Diabetes, %	19.3	17.5	19.8	0.15	19.4	19.3	0.96
Heart failure, %	5.7	5.4	5.8	0.73	5.7	5.6	0.88
CAD, %	9.6	8.9	9.8	0.44	8.9	11.5	<0.05
Prior stroke/TIA, %	2.8	3.7	2.5	0.09	2.8	2.5	0.59
PAD, %	3.0	2.5	3.2	0.31	3.0	3.2	0.73
COPD, %	7.4	3.6	8.2	<0.0001	7.2	7.7	0.58
Renal function							
Known renal disease, %	3.3	2.7	3.4	0.34	3.2	3.5	0.70
Microalbuminuria, %	6.5	5.7	6.7	0.53	6.6	6.0	0.65

Legend: *AZL*-*M* azilsartan medoxomil, *ACE* angiotensin-converting enzyme, *SBP* systolic blood pressure, *DPB* diastolic blood pressure, *CAD* coronary artery disease, *TIA* transient ischaemic attack, *PAD* peripheral artery disease. Values are indicated in percent (%), median (interquartile range), or mean ± standard deviation

The 12 month follow-up period was completed by a total of 3 082 (80.1 %) patients, including 2 237 in the AZL-M group and 845 in the ACE-inhibitor group (Fig. 1). In the group that did not complete the follow-up, slightly fewer patients were female, had COPD, and the average body weight was higher. There were no other differences in baseline characteristics between the patients that did and did not complete the follow-up.

### Achievement of BP targets based on recent national and international guidelines

**Fig. 2:**
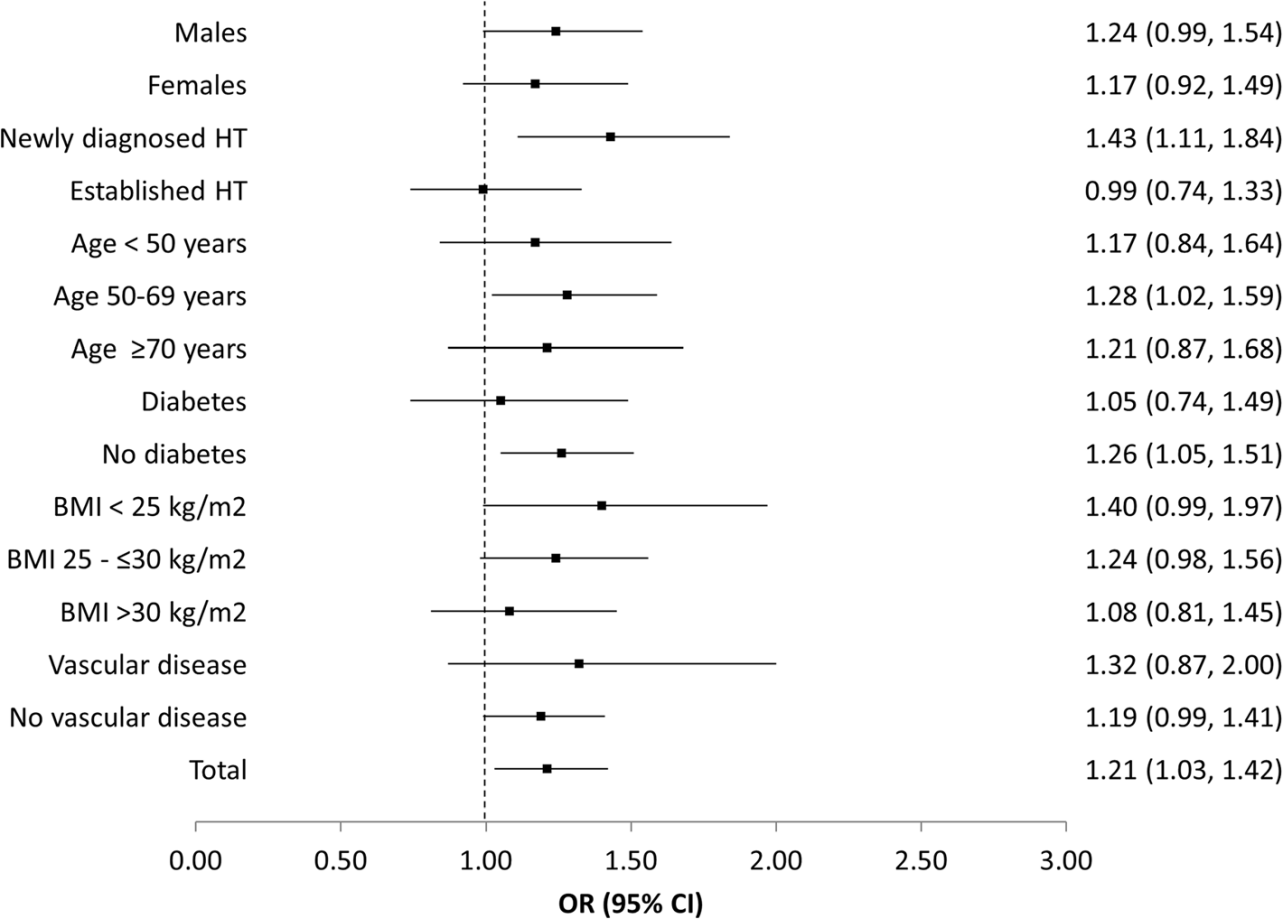
AZL-M vs. ACE-inhibitors in patients with a 12 month follow-up–target BP achievement (<140/90 mmHg). Legend: HT, hypertension; BMI, body mass index; target BP achievement is defined as an SBP of <140 mmHg and a DBP of <90 mmHg

Blood pressure values achieved at 12 months were 134.1 ± 12.9 mmHg / 80.8 ± 8.0 mmHg for AZL-M and 134.9 ± 13.1 mmHg / 81.4 ± 8.7 mmHg for the ACE-inhibitor group (*p* = 0.11 and *p* = 0.07, respectively; Additional file 1: Table S1). Using raw unadjusted data for patients who completed the 12 month follow-up, mean reductions in SBP and DBP in the AZL-M group (25.9 and 13.0 mmHg, respectively) were greater than those recorded for the ACE-inhibitor group (22.6 and 11.4 mmHg, respectively; *p* < 0.0001 and < 0.001, respectively). Accordingly, the proportion of patients who attained the target BP level of <140/90 mmHg was greater in the AZL-M group (61.1 %) compared with the ACE-inhibitor group (56.4 %; *p* < 0.05; OR, 1.21; 95 % CI, 1.03–1.42; Table 2) overall and in subgroups of patients (Fig. 2). Following adjustment for baseline SBP/DBP (model 1), and for baseline SBP/DBP, newly diagnosed or established hypertension, age, gender, and the presence of diabetes (model 2), compared with ACE-inhibitor treatment, AZL-M treatment was still associated with statistically significant reductions in SBP (*p* < 0.05 and 0.01 for the two respective models) and DBP (*p* < 0.05 for both models). Furthermore, there was a greater proportion of patients who achieved a BP of <140/90 mmHg (*p* < 0.01 for both models). The respective analyses for the 6 months follow-up are displayed in Additional file 1: Table S1 and Additional file 2: Table S2, which show no major differences compared to the 12 months analysis.

**Table 2:** Blood pressure reductions-comparison of treatment groups in patients with a 12 months follow-up

	differences at 12 months vs. baseline		
	AZL-M(*n* = 2 237)Δ value (95 % CI)	ACE-inhibitor(*n* = 845) Δ value (95 % CI)	*p*-value for the comparisonof differences vs. baseline
Raw (unadjusted)			
∆ SBP, mmHg	25.9 (25.1–26.7)	22.6 (21.3–23.8)	<0.0001
∆ DBP, mmHg	13.0 (12.5–13.5)	11.4 (10.6–12.2)	<0.001
∆ Mean BP, mmHg	17.3 (16.7–17.8)	15.1 (14.3–15.9)	<0.0001
∆ Pulse pressure, mmHg	12.9 (12.2–13.6)	11.1 (10.1–12.2)	<0.05
∆ Heart rate, bpm	3.0 (2.6–3.5)	2.9 (2.2–3.7)	0.64
B*P* < 140/90 mmHg, %	61.1 (59.0–63.1)	56.4 (53.0–59.8)	<0.05
Model 1 (adjusted)			
∆ SBP, mmHg	25.3 (24.7–25.8)	24.1 (23.3–25.0)	<0.05
∆ DBP, mmHg	12.7 (12.4–13.1)	12.0 (11.5–12.5)	<0.05
∆ Mean BP, mmHg	17.0 (16.6–17.2)	16.0 (15.5–16.6)	<0.05
∆ Pulse pressure, mmHg	12.5 (12.1–13.0)	12.1 (11.4–12.9)	0.39
∆ Heart rate, bpm	3.1 (2.7–3.4)	2.9 (2.4–3.4)	0.57
B*P* < 140/90 mmHg, %	61.4 (59.4–63.4)	55.9 (52.5–59.2)	<0.01
Model 2 (adjusted)			
∆ SBP, mmHg	25.3 (24.8–25.8)	24.0 (23.1–24.8)	<0.01
∆ DBP, mmHg	12.7 (12.4–13.1)	12.0 (11.5–12.5)	<0.05
∆ Mean BP, mmHg	17.0 (16.6–17.3)	16.0 (15.4–16.5)	<0.01
∆ Pulse pressure, mmHg	12.6 (12.1–13.0)	12.0 (11.2–12.7)	0.15
∆ Heart rate, bpm	3.1 (2.7–3.4)	2.8 (2.3–3.4)	0.50
B*P* < 140/90 mmHg, %	61.7 (59.6–63.7)	55.5 (52.1–58.9)	<0.01

Legend: *AZL*-*M* azilsartan medoxomil, *ACE* angiotensin-converting enzyme, *SBP* systolic blood pressure, *DBP* diastolic blood pressure. To illustrate the adjusted changes in BP, 3 pretreatment BP values were chosen representing the three borders between four quartiles; model 1: adjusted for SBP/DBP at baseline; model 2: adjusted for SBP/DBP at baseline (model 1), newly diagnosed or established hypertension, age, gender, and diabetes

The subgroups of patients with newly diagnosed hypertension, aged 50–69 years, and without diabetes were found to be statistically more likely to achieve target BP when treated with AZL-M rather than an ACE-inhibitor; and the results were significant (Fig. 2). In contrast, gender, body mass index (BMI), and the presence of vascular disease did not significantly affect the outcome.

### Safety profile

**Fig. 3:**
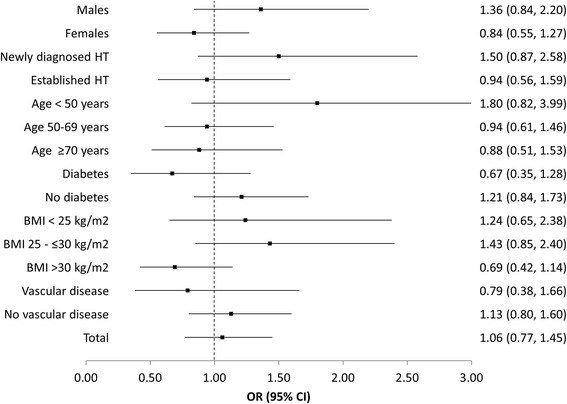
AZL-M vs. ACE-inhibitors in patients with a 12 month follow-up–any AE. Legend: HT, hypertension; BMI, body mass index

In terms of the safety of AZL-M, there was no difference in the proportion of patients experiencing an AE in comparison to those being treated with an ACE-inhibitor (*p* = 0.73; Table 3); however, a higher percentage of the AZL-M group died (10/2 237; 0.4 % vs. 1/845; 0.1 %). Causes of death were myocardial infarction (2×), post-procedural sepsis (1×), prostate cancer (1×), pancreatic cancer (1×), pneumonia (1×), a road traffic accident (1×), and unknown (3×) in the AZL-M group and unknown (1×) in the ramipril group. For one patient in the AZL-M group that died of an unknown cause there was uncertainty about the causal relationship between AZL-M treatment and death. On analysis of various patient subgroups, no differences in the incidence of AEs were apparent between the AZL-M and ACE-inhibitor groups (Fig. 3). Furthermore, there were no significant differences in laboratory values between the groups, including HbAc1, fasting glucose, creatinine, potassium, and estimated glomerular filtration rate (eGFR).

**Table 3:** Safety of AZL-M and ACE-inhibitors during 12 month follow-up

	AZL-M		ACE-inhibitor		*p*-value
	(*n* = 2 237)		(*n* = 845)		
	mean ± SD or %		mean ± SD or %		
Patients without an AE, %	92.9		93.3		0.73
Patients with an AE, %	7.1		6.7		0.73
Laboratory values	Baseline AZL-M	∆ at 12 months FU	Baseline ACEi	∆ at 12 months FU	*p*-value ∆ AZL-M vs. ∆ ACEi
HbA1c, %	5.9 ± 1.2	0.01 ± 0.97	5.9 ± 1.3	0.08 ± 0.95	0.94
Fasting glucose, mg/dl	100.0 ± 27.0	3.00 ± 57.35	101.3 ± 24.8	1.39 ± 18.35	0.56
Creatinine, mg/dl	1.2 ± 0.8	−0.02 ± 0.82	1.3 ± 1.0	−0.05 ± 0.82	0.17
Potassium, mmol/l	4.1 ± 0.5	0.02 ± 0.47	4.1 ± 0.5	0.03 ± 0.50	0.70
eGFR, ml/min/1.73 m^2^	62.3 ± 18.7	−1.79 ± 12.61	64.0 ± 20.5	−0.24 ± 13.15	0.23

Legend: *AZL*-*M* azilsartan medoxomil, *ACE* angiotensin-converting enzyme, *AE* adverse event, *HbA*1*c* glycated haemoglobin, *eGFR* estimated glomerular filtration rate

### Treatment persistence

**Fig. 4:**
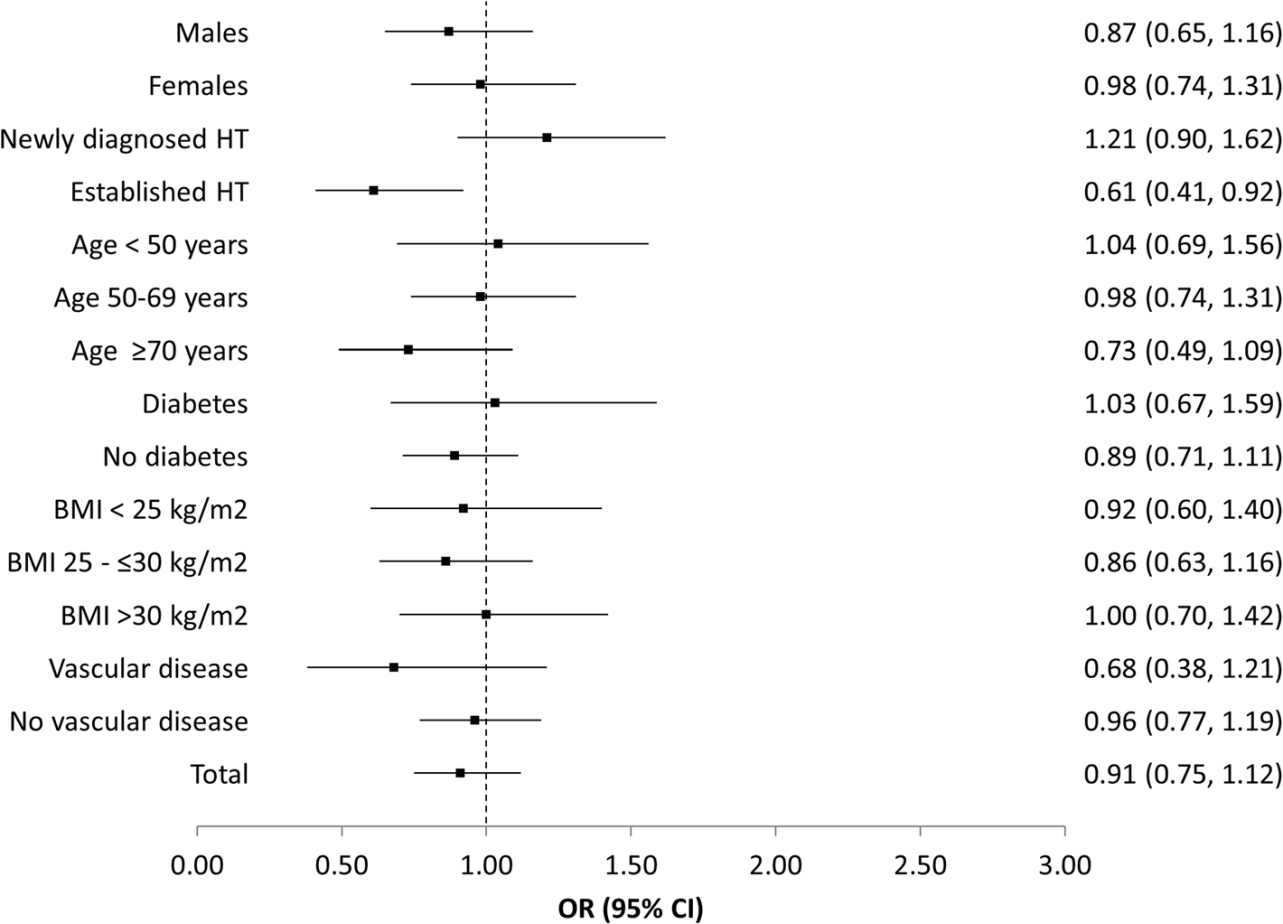
AZL-M vs. ACE-inhibitors in patients with a 12 month follow-up–no treatment target adjustment. Legend: HT, hypertension; BMI, body mass index

There was no evident difference between the likelihood of patients taking AZL-M or an ACE-inhibitor in terms of a requirement for treatment adjustment during the 12 month follow-up period (OR, 0.91; 95 % CI, 0.75–1.12; Fig. 4). However, the patients with established hypertension were seen to be more likely to need a change in treatment if they were being treated with an ACE-inhibitor (OR, 0.61; 95 % CI, 0.41–0.92).

## Discussion

In the present study, the efficacy, safety, and tolerability of antihypertensive monotherapy using either AZL-M or an ACE-inhibitor was evaluated in real life clinical practice. The key finding was that after 12 months of treatment, both reductions in SBP and DBP, and the percentage of patients who attained target BP levels, were significantly greater with AZL-M treatment compared to that with an ACE-inhibitor.

### Efficacy outcomes in perspective

On analysis of the raw data, it was seen that the mean reductions in SBP and DBP were greater in the AZL-M group (∆25.9 mmHg) relative to the ACE-inhibitor group (∆22.6 mmHg), with an additional 4.7 % of patients reaching the target level of BP control. Similar results were obtained after adjusting for baseline SBP/DBP (model 1), and SBP/DBP, newly diagnosed or established hypertension, age, gender, and diabetes (model 2). This is in principal agreement with the data reported by Bönner et al. and who demonstrated improved BP reduction for patients who were allocated AZL-M compared to ramipril in a randomised trial [13]. In the Bönner trial, the primary efficacy endpoint was the change in clinic trough, seated systolic BP from baseline with ambulatory BP additionally provided. AZL-M 40 and 80 mg reduced both clinic systolic BP and mean ambulatory systolic BP significantly more than ramipril at a dose of 10 mg (clinic SBP −20.6 ± 0.9 with 40 mg and −21.2 ± 0.9 with 80 mg AZL-M vs.-12.2 ± 0.9 with ramipril; *p* < 0.001 for both doses). Compared to these numbers differences in the present trial were low, with 1.3 mmHg systolic (*p* < 0.01) and 0.7 mmHg diastolic (*p* < 0.05) after adjustment (model 2). The pronounced BP lowering effect in the Bönner RCT compared to the observational study may be the result of the selection criteria that were applied in the RCT, resulting in many patients treated in clinical practice, being excluded from the randomized trial, for which it has been shown to have a lesser effect [19]. The results are nonetheless important since a number of analyses have shown that (even small) changes in blood pressure will result in a linear reduction in morbidity and mortality [20].

Furthermore, Bönner [13] found that the difference in blood pressure lowering remained for each of the subgroups analysed, which included age, gender, BMI, clinic SBP, and eGFR. In the present registry, no difference in the rate of target BP achievement was found in the gender and BMI subgroups; however, patients with newly diagnosed hypertension, those aged 50–69 years, and those without diabetes were more likely to reach the target value if treated with AZL-M rather than an ACE-inhibitor.

The efficacy of AZL-M versus ACE-inhibitors for reducing BP may be explained by the different biochemical characteristics and mechanisms of action of the two drug classes. ACE-inhibitors work via competitive inhibition of the enzyme that is responsible for the conversion of angiotensin I to angiotensin II. However, angiotensin II can also be produced via other mechanisms; therefore, such a drug cannot completely inhibit its formation [4, 21]. ARBs, on the other hand, work as antagonists for the AT1 angiotensin II receptor, providing a more direct and complete inhibition of the BP raising effects of the RAS. Indeed, the results of the ONTARGET trial demonstrated a greater reduction in BP for patients treated with the ARB telmisartan in comparison to Ramipril [22]. Another study showed the equivalent efficacy of valsartan and lisinopril [23]. As AZL-M has demonstrated higher efficacy for BP lowering in comparison to other sartans, it is unsurprising that it has performed better in the patients included in the EARLY registry [4, 9, 11, 24].

### Safety outcomes in perspective

In the present registry, no significant difference in the incidence of AEs was observed between the AZL-M and ACE-inhibitor groups, indicating an equivalent level of safety. Furthermore, there were no apparent differences in laboratory values between the two groups. On analysis of the subgroups, again no disparity was found in the rate of AEs with the two treatment populations. This is in agreement with a number of other studies comparing the safety of ARBs with that of ACE-inhibitors. Roy et al. evaluated the incidences of death, stroke, CAD, and chronic kidney disease in a population of hypertensive patients being treated with one of either class of drug, and found no significant differences between the two groups [8]. Li et al. and Reboldi et al. reviewed the available literature regarding comparisons between ACE-inhibitors and ARBs and both concluded that there were no significant differences between the two drug categories in terms of total mortality risk, cardiovascular risk, and cardiovascular mortality [6, 25]. Hasvold et al. found a similar risk of cardiovascular disease in patients being treated with the ACE-inhibitor enalapril and the ARB candesartan [7], while Bönner et al. compared ramipril with AZL-M and also found no significant variations in the occurrence of AEs between the two groups, although they reported a slightly higher occurrence of cough and lower incidences of back pain and dizziness in the ramipril patients [13]. An observational study reported by Petrella et al. compared the tolerability of ARBs to other antihypertensive medications, and found that patients treated with the former were less likely to experience a cardiovascular event [26]. These data demonstrate the favourable safety profile of both classes of drug.

### Limitations

There were a number of limitations to the present registry. Firstly, owing to the inherent characteristics of an observational study, treatment allocation was not randomised and thus patient characteristics and BP values at baseline not comparable. This resulted in an imbalance in patient number between the two groups, although the sizes of both were high overall. Furthermore, ramipril was the most commonly prescribed ACE-inhibitor, and while a limited number of others were allowed, the numbers of patients were not high enough to draw comparisons between them. Another limitation is that the medication regime was left to the discretion of the treating physician, which may have resulted in patients with certain characteristics being preferentially prescribed one class of drug over the other. This would likely introduce a level of bias to the data. Finally, the incidence of AEs was extremely low in both patient groups, making it difficult to satisfactorily determine differences between the two classes of drug. AEs further were tracked more closely in the AZL-M arm, giving rise to the speculation that rates in the ACEi arm are actually higher than reported.

## Conclusions

In conclusion, data from this study may help to inform clinical decisions as to which is the most appropriate RAS-targeted antihypertensive agent. The relative impact of ACE-inhibitors versus ARBs on the rate of specific cardiovascular events and other clinically relevant endpoints is yet to be resolved. However, this study adds to the body of literature suggesting that when BP is taken as the sole measure of the efficacy of these drugs, AZL-M is more effective than ACE-inhibitors. Moreover, this study indicates that data from clinical trials of AZL-M may be extrapolated to real life clinical practice.

## Additional files

Additional file 1: Table S1. Blood pressure values achieved at 6 and 12 months respectively (DOCX 28 kb) Additional file 2: Table S2. Blood pressure reductions-comparison of treatment groups in patients with a 6 months follow-up (DOCX 29 kb)

## Abbreviations

ACE: angiotensin converting enzyme
ACEi: angiotensin converting enzyme inhibitor
ARB: angiotensin receptor blocker
AZL-M: azilsartan medoxomil
BP: blood pressure
DBP: diastolic blood pressure
EARLY: treatment with Azilsartan compared to ACE-inhibitors in anti-hypertensive therapy
RCT: randomized controlled trial
SBP: systolic blood pressure
SD: standard deviation
