# Supplementary material for: Azilsartan compared to ACE inhibitors in anti-hypertensive therapy: one-year outcomes of the observational EARLY registry
Source: BMC Cardiovasc Disord. 2016 Mar 8;16:56. doi: 10.1186/s12872-016-0222-6 (PMC4784379; doi:10.1186/s12872-016-0222-6)
Supplement: Additional file 1: Table S1. — Blood pressure values achieved at 6 and 12 months respectively (DOCX 28 kb) [file 12872_2016_222_MOESM1_ESM.docx]

## Additional 1: Table S1 - Blood pressure values achieved at 6 and 12 months respectively

|  | BP values achieved at 6 months | | | BP values achieved at 12 months | | |
| --- | --- | --- | --- | --- | --- | --- |
|  | AZL-M  (n = 2 237)  mean±SD | ACE-inhibitor  (n = 845)  mean±SD | p-value for the comparison of BP values achieved | AZL-M  (n = 2 237)  mean±SD | ACE-inhibitor  (n = 845)  mean±SD | p-value for the comparison of BP values achieved |
| Raw (unadjusted) |  |  |  |  |  |  |
| SBP, mmHg | 135.4±13.8 | 136.0±14.1 | 0.14 | 134.1±12.9 | 134.9±13.1 | 0.11 |
| DBP, mmHg | 81.6±8.6 | 82.1±8.7 | 0.21 | 80.8±8.0 | 81.4±8.7 | 0.07 |
| Mean BP, mmHg | 99.5±8.9 | 100.1±9.2 | 0.15 | 98.6±8.3 | 99.2±8.8 | 0.06 |
| Pulse pressure, mmHg | 53.8±12.0 | 54.0±12.0 | 0.54 | 53.3±11.4 | 53.5±11.6 | 0.78 |
| Heart rate, bpm | 73.5±8.5 | 74.1±8.7 | 0.06 | 73.4±8.1 | 73.7±8.3 | 0.22 |

Legend: AZL-M, azilsartan medoxomil; ACE, angiotensin-converting enzyme; SBP, systolic blood pressure; DBP, diastolic blood pressure. To illustrate the adjusted changes in BP, 3 pretreatment BP values were chosen representing the three borders between four quartiles
