# Supplementary material for: Azilsartan compared to ACE inhibitors in anti-hypertensive therapy: one-year outcomes of the observational EARLY registry
Source: BMC Cardiovasc Disord. 2016 Mar 8;16:56. doi: 10.1186/s12872-016-0222-6 (PMC4784379; doi:10.1186/s12872-016-0222-6)
Supplement: Additional file 2: Table S2. — Blood pressure reductions-comparison of treatment groups in patients with a 6 months follow-up (DOCX 29 kb) [file 12872_2016_222_MOESM2_ESM.docx]

## Additional file 2: Table S2. Blood pressure reductions - comparison of treatment groups in patients with a 6 months follow-up

|  | differences at 6 months vs. baseline | | |
| --- | --- | --- | --- |
|  | AZL-M  (n = 2 237)  Δ value (95%CI) | ACE-inhibitor  (n = 845)  Δ value (95%CI) | p-value for the comparison of differences |
| Raw (unadjusted) |  |  |  |
| ∆ SBP, mmHg | 24.7(23.9-25.5) | 21.4(20.1-22.7) | <0.0001 |
| ∆ DBP, mmHg | 12.3(11.8-12.8) | 10.8(10.0-11.6) | < 0.01 |
| ∆ Mean BP, mmHg | 16.4(16.0-16.9) | 14.3(13.5-16.2) | <0.0001 |
| ∆ Pulse pressure, mmHg | 12.4(11.8-13.1) | 10.7(9.5-11.8) | < 0.05 |
| Heart rate, bpm | 3.0(2.6-3.5) | 2.6(1.9-3.3) | 0.23 |
| BP <140/90 mmHg, % | 56.3(54.3-58.4) | 52.5(49.1-55.9) | 0.06 |
| Model 1 (adjusted) |  |  |  |
| ∆ SBP, mmHg | 24.1(23.5-24.7) | 23.0(22.1-23.9) | <0.05 |
| ∆ DBP, mmHg | 12.0(11.7-12.4) | 11.4(10.8-12.0) | 0.06 |
| ∆ Mean BP, mmHg | 16.1(15.7-16.4) | 15.3(14.7-15.9) | <0.05 |
| ∆ Pulse pressure, mmHg | 12.1(11.6-12.6) | 11.6(10.8-12.4) | 0.30 |
| ∆ Heart rate, bpm | 3.0(2.7-3.4) | 2.5(2.0-3.1) | 0.12 |
| BP <140/90 mmHg, % | 56.8(54.7-58.9) | 51.6(48.1-55.0) | < 0.05 |
| Model 2 (adjusted) |  |  |  |
| ∆ SBP, mmHg | 24.1(23.6-24.7) | 22.8(22.0-23.8) | <0.05 |
| ∆ DBP, mmHg | 12.0(11.7-12.4) | 11.4(10.9-12.0) | 0.10 |
| ∆ Mean BP, mmHg | 16.1(15.7-16.4) | 15.2(14.6-15.8) | <0.05 |
| ∆ Pulse pressure, mmHg | 12.1(11.7-12.6) | 11.4(10.6-12.2) | 0.12 |
| ∆ Heart rate, bpm | 3.0(2.7-3.4) | 2.5(2.0-3.1) | 0.11 |
| BP <140/90 mmHg, % | 56.9(54.8-59.0) | 51.4(48.0-54.8) | < 0.01 |

Legend: AZL-M, azilsartan medoxomil; ACE, angiotensin-converting enzyme; SBP, systolic blood pressure; DBP, diastolic blood pressure. To illustrate the adjusted changes in BP, 3 pretreatment BP values were chosen representing the three borders between four quartiles; model 1: adjusted for SBP/DBP at baseline; model 2: adjusted for SBP/DBP at baseline (model 1), newly diagnosed or established hypertension, age, gender, and diabetes.
